# Identification of disulfidptosis- and ferroptosis-related transcripts in periodontitis by bioinformatics analysis and experimental validation

**DOI:** 10.3389/fgene.2024.1402663

**Published:** 2024-07-09

**Authors:** Yu Fu, Tingrui Xu, Mengru Guo, Wenhao Lv, Ning Ma, Li Zhang

**Affiliations:** Hospital of Stomatology, Jilin University, Changchun, China

**Keywords:** bioinformatics, disulfidptosis, ferroptosis, periodontitis, immune infiltration, machine learning

## Abstract

**Background:**

Disulfidptosis and ferroptosis are forms of programmed cell death that may be associated with the pathogenesis of periodontitis. Our study developed periodontitis-associated biomarkers combining disulfidptosis and ferroptosis, which provides a new perspective on the pathogenesis of periodontitis.

**Methods:**

Firstly, we obtained the periodontitis dataset from public databases and found disulfidptosis- and ferroptosis-related differentially expressed transcripts based on the disulfidptosis and ferroptosis transcript sets. After that, transcripts that are tissue biomarkers for periodontitis were found using three machine learning methods. We also generated transcript subclusters from two periodontitis microarray datasets: GSE16134 and GSE23586. Furthermore, three transcripts with the best classification efficiency were further screened. Their expression and classification efficacy were validated using qRT-PCR. Finally, periodontal clinical indicators of 32 clinical patients were collected, and the correlation between three transcripts above and periodontal clinical indicators was analyzed.

**Results:**

We identified six transcripts that are tissue biomarkers for periodontitis, the top three transcripts with the best classification, and delineated two expression patterns in periodontitis.

**Conclusions:**

Our study found that disulfidptosis and ferroptosis were associated with immune responses and may involve periodontitis genesis.

## 1 Introduction

Periodontitis is an inflammatory, destructive disease involving periodontal supporting tissues due to dysbiosis of the oral microbiota, causing interaction with the host immune defence system (Kinane et al., 2017). According to the analysis of the Global Burden of Disease Study 1990-2019, periodontitis, the sixth most prevalent disease in the world, has a prevalence of 50%, with 1.1 billion people suffering from severe periodontitis (Chen et al., 2021; Kassebaum et al., 2014). Loss of connective and bone tissue caused by periodontitis is the leading cause of tooth loss in adults (Pihlstrom et al., 2005). On the one hand, tooth loss and chewing dysfunction in patients with periodontitis may negatively affect their nutritional intake, quality of life, and even mental health (Reynolds and Duane, 2018). On the other hand, periodontal tissue inflammation may increase the body’s overall inflammatory burden, contributing to further deterioration of diseases such as diabetes and cardiovascular diseases (Preshaw and Bissett, 2019; Sanz et al., 2020). For this reason, much research has been done on the mechanisms of periodontitis over a long period. However, the exact pathogenesis of this disease remains unclear. It is widely recognized that a highly inflammatory state, triggered by ecological dysregulation of oral microorganisms and excessive host immune response to the microorganisms, is closely related to the pathogenesis of periodontitis (Kinane et al., 2007; Hajishengallis and Lamont, 2012; Roberts and Darveau, 2015). Notably, multiple forms of cell death have been found to play an essential role in developing inflammation and mediating immune responses (Weindel et al., 2022). Various forms of cell death have also emerged as a new hotspot for investigating the mechanisms of chronic inflammatory diseases such as periodontitis. Therefore, the investigation of the underlying mechanisms of periodontitis, especially the cell death and immuno-inflammatory regulatory mechanisms, will provide new insights into the pathogenesis of periodontitis.

Disulfidptosis is a new form of cell death, usually caused by abnormal accumulation of disulfides leading to disulfide stress, which disrupts the cytoskeleton (Liu et al., 2023). In addition, the reduced form of nicotinamide adenine dinucleotide phosphate (NADPH) provides the reducing ability to prevent disulfide stress critical for maintaining cell survival against disulfidptosis (Zheng et al., 2023). First, we hypothesized that the cell’s redox state is associated with the onset of disulfidptosis. Numerous studies have shown that periodontitis is an inflammatory disease caused by oxidative stress in which the oxidant and antioxidant systems are imbalanced. For example, excessive reactive oxygen species (ROS) are produced by overactivated polymorphonuclear neutrophils in dental plaque, and excessive ROS leads to an increased oxidant load, which results in oxidative stress in the tissues and triggers periodontal tissue destruction (Sczepanik et al., 2020). Second, studies have shown a significant correlation between the severity of periodontitis and serum levels of disulfides, which are significantly elevated in patients with periodontitis compared to the healthy population (Tayman et al., 2021). Another study also confirmed that thiols with reducing properties were significantly reduced in gingival tissues of periodontitis patients (Cerkezi et al., 2024), and that this reducing compound plays a crucial role in resistance to oxidative stress. Furthermore, disulfidptosis has been progressively studied in inflammatory diseases such as obligatory myelitis and ulcerative colitis (Li et al., 2024; Xiong et al., 2024). Thus, the state of oxidative stress in periodontitis and the changes in disulfide levels allow us to reasonably hypothesize that the development of periodontitis may be associated with disulfidptosis.

Ferroptosis is a type of iron-dependent regulatory cell death. It affects glutathione peroxidase, leading to decreased cellular antioxidant capacity, ROS accumulation, and lipid peroxidation, which results in oxidative cell death and tissue damage (Stockwell et al., 2017). Iron-dependent oxidative stress and lipid peroxidation are recognized as critical mechanisms of ferroptosis in periodontitis (Chen et al., 2022). Regarding the first aspect, iron concentration fluctuations correlate with the severity of periodontitis (Boyer et al., 2018). Periodontal pathogenic microorganisms sequester iron from iron-containing compounds such as ferritin and hemoglobin in infected periodontal tissues to support their growth and reproduction (Olczak et al., 2005). Excess free iron creates an iron overload, which triggers the Fenton reaction, producing substantial amounts of ROS (Lewis, 2010; Ke et al., 2017). Beyond ROS generated by iron overload, human immune defence cells, such as polymorphonuclear leukocytes, consume NADPH to produce ROS (Hirschfeld et al., 2017). The overproduction of ROS results in oxidative damage to proteins and lipid peroxidation in periodontal tissues, closely associated with the severity of periodontitis (Chiu et al., 2017). From the second perspective, by-products of lipid peroxidation are significantly elevated in patients with chronic periodontitis, and indicators such as glutathione peroxidase (GPX) and the glutathione/oxidized glutathione ratio are altered (Borges et al., 2007; Fentoğlu et al., 2015). Hypoxia in periodontal pockets leads to the high expression of hypoxia-inducible factor-1 (HIF-1), causing fatty acid deposition in the microenvironment (Ezzeddini et al., 2021). ROS attacks lipids, such as phospholipids, in periodontal tissues, resulting in oxidative damage to lipids, contributing to periodontitis’s progression. In addition, lipid peroxidation is a chain reaction that leads to biofilm damage and altered fluidity (Halliwell and Chirico, 1993). In summary, the correlation between the development of periodontitis and ferroptosis is well-supported.

Multiple forms of cell death can coexist and interact within the context of periodontitis. The roles of cell death modalities such as autophagy, pyroptosis, and necroptosis in periodontitis have been extensively studied. However, the interplay between ferroptosis and disulfidptosis in periodontitis remains underexplored. Our findings suggest that an imbalance in the redox state not only negatively impacts the reduction state preventing disulfide stress, thereby linking to disulfidptosis, but is also closely associated with the mechanism of ferroptosis. Further exploration is required to learn the specific mechanisms of disulfidptosis and ferroptosis in periodontitis. In our study, we developed transcriptional biomarkers related to periodontitis that integrate disulfidptosis and ferroptosis. These biomarkers provide new insight into periodontitis pathogenesis’s molecular mechanisms and help explore innovative diagnostic methods for periodontitis.

## 2 Methods

### 2.1 Data acquisition and differential expression analysis

We acquired three independent periodontitis microarray datasets (GSE16134, GSE23586, and GSE10334) from the GEO database utilizing the “GEOquery” package in R software (version 4.2.2). Each dataset was based on the microarray platform GPL570. These raw data were subsequently normalized and preprocessed. To enhance study reliability, datasets GSE16134 and GSE23586 were merged to form a training set comprising 244 periodontitis and 72 control samples. Batch effects were removed from the merged datasets utilizing R software’s “limma” and “sva” packages. Dataset GSE10334 served as the validation set, containing 183 periodontitis and 64 healthy samples.

Ferroptosis-related transcripts were sourced from the FerrDb website (http://www.zhounan.org/ferrdb/) (Zhou and Bao, 2020), yielding 259 ferroptosis-related transcripts. Disulfidptosis-related transcripts were acquired from previous literature (Liu et al., 2023) and the MSigDB website (https://www.gsea-msigdb.org/gsea/msigdb/), resulting in a total of 23 disulfidptosis-related transcripts. Pearson correlation coefficients for ferroptosis and disulfidptosis transcript expression in the training set were computed using “cor.test ()” in R software, setting thresholds at “|cor| > 0.3 and *p* < 0.05” to identify disulfidptosis- and ferroptosis-related transcripts. This study did not use a direct crossover of the 2 cell death transcript sets to identify DFR transcripts. While direct crossover is a straightforward method, it can potentially overlook some biologically essential transcripts. This correlation coefficient approach has been widely employed in bioinformatics (Jia et al., 2024).

Differential expression analysis of the previously identified transcripts utilized the "limma" R package. Transcripts exhibiting *p*-values <0.05 and |log2 (fold change)| > 0.5 were identified as differentially expressed disulfidptosis- and ferroptosis-related transcripts.

### 2.2 Identification of molecular mechanisms

To elucidate the biological functions of the differentially expressed disulfidptosis- and ferroptosis-related transcripts, Gene Ontology (GO) and Kyoto Encyclopedia of Genes and Genomes (KEGG) analysis were performed. The study was completed utilizing the “clusterProfiler” R package. A protein-protein interaction (PPI) network of the protein products of the differentially expressed disulfidptosis- and ferroptosis-related transcripts was constructed using the STRING online platform (https://www.string-db.org), with a minimum interaction threshold set at 0.4. The interaction results were visualized utilizing Cytoscape software (version 3.9.1). The top ten most highly associated hub protein products of transcripts in the PPI network were identified using the MCC algorithm in CytoHubba, a plugin within Cytoscape.

### 2.3 Machine learning

Three machine learning methods were employed to screen transcriptional biomarkers in differentially expressed disulfidptosis- and ferroptosis-related transcripts for periodontitis: the least absolute shrinkage and selection operator (LASSO), support vector machine (SVM), and random forest (RF). They were implemented utilizing the “glmnet”, “e1071”, and “randomForest” R packages, respectively. Transcripts identified by intersecting the results of these three machine learning methods were defined as disulfidptosis- and ferroptosis-related transcripts that are tissue biomarkers for periodontitis.

### 2.4 Construction of disulfidptosis- and ferroptosis-related transcripts classification model and nomogram model

Logistic regression analysis was utilized to evaluate the capacity of the machine learning screened transcriptional biomarkers to differentiate between periodontitis and healthy tissues to develop a classification model for periodontitis. This model allowed us to obtain a risk score for each patient using the formula risk score = ∑ (Expi × β_i_). In this formula, β_i_ represents the risk coefficient of each transcriptional biomarker, and Expi represents the transcript’s expression. On this basis, receiver operating characteristic (ROC) analysis was further performed using the “pROC” R package to evaluate the prediction model’s classification ability. Three transcriptional biomarkers with optimal classification ability were screened based on the area under the curve (AUC). In addition, we plotted a nomogram based on the transcriptional biomarkers screened by machine learning. This model aids in understanding the contribution of each signature to the disease and accurately predicts disease risk. Calibration curves, decision risk curves (DCA), and clinical impact curves (CIC) help us evaluate the nomogram’s effectiveness. Finally, we used an external dataset, GSE10334, as a validation set.

### 2.5 Immune infiltration analysis

We employed the CIBERSORT method, as detailed in the reference (Chen et al., 2018). This method analyzes the proportion of 22 immune cells infiltrating complex tissues. We calculated the proportion of each immune cell infiltration in gingival tissues from periodontitis patients and healthy individuals. We also showed the difference between each cell in the two groups. More importantly, we calculated the Spearman correlation coefficients between transcriptional biomarkers and the 22 types of infiltrating immune cells. This helps us to understand the role of disulfidptosis and ferroptosis in periodontitis further.

### 2.6 Consensus clustering analysis of disulfidptosis- and ferroptosis-related transcripts transcript clusters

Utilizing the expression levels of transcriptional biomarkers, the periodontitis samples in the training set underwent consensus clustering analysis using the “Consensus Cluster Plus” package in R software. The k-means clustering method was employed for 50 iterations. Principal component analysis (PCA) validated the clustering’s reliability. Additionally, immune infiltration analysis was conducted to investigate the variations in immune cell infiltration among the subclusters. Gene Set Enrichment Analysis (GSEA) and Gene Set Variation Analysis (GSVA) were employed to elucidate the functional disparities between subclusters identified in the prior cluster analysis.

### 2.7 Collection of clinical samples

Clinical samples were collected to validate the study’s reliability. During crown lengthening surgery, healthy gingival tissues were collected from 16 individuals without periodontitis. Additionally, gingival tissues were collected from 16 patients with periodontitis during gingivectomy. The periodontal conditions of all patients were assessed by the same periodontist specializing in periodontology, who recorded clinical indicators such as probing depth (PD), attachment loss (AL), gingival index (GI), and plaque index (PI). All gingival samples were sourced from Jilin University Stomatological Hospital. Samples were promptly transferred to tissue RNA preservation solution (RNAwait) (Solarbio, SR0020, Beijing, CHINA) overnight at 4°C, followed by −80°C storage for future experimental use. All subjects in the disease groups were diagnosed with periodontitis based on the American Academy of Periodontology 1999 classification. Inclusion and exclusion criteria for this study are detailed in Supplementary Material S1, while periodontal clinical indicators and participant assessment criteria are outlined in Supplementary Material S2. All subjects have given informed consent. This study received approval from the Ethics Committee of Jilin University Stomatological Hospital (approval number JDKQ202343), adhering to the Declaration of Helsinki of 1964, its subsequent amendments, and ethical standards.

### 2.8 Quantitative real-time PCR

First, total RNA was extracted from the gingival tissues of the disease group and the control group using the Trizol method. Second, the total RNA of the samples was quantified using a NanoDrop 2000 analyzer. Third, the mRNA was converted into cDNA using the 1st Strand cDNA Synthesis SuperMix for qPCR (YEASEN, 11141ES60-100T, Shanghai, China). Fourth, qRT-PCR was performed on a Bio-Rad analyzer using primers for the top three transcripts with the best classification (Supplementary Material S3) and the qPCR SYBR Green Master Mix (YEASEN, 11202ES08, Shanghai, China). GAPDH was used as the internal reference transcript in this experiment. Fifth, using the 2^−ΔΔCt^ method, the results from the analyzer were calculated to determine the relative mRNA expression levels of the top three transcripts with the best classification. This experiment was repeated three times to ensure its reliability. Additionally, we calculated Pearson’s correlation coefficients between the top three transcripts with the best classification expression in 16 periodontitis samples. ROC curves for 32 study cases from Jilin University Stomatological Hospital were plotted to verify the efficacy of the top three transcripts with the best classification in distinguishing periodontitis gingival tissues from healthy ones.

### 2.9 Association analysis of transcriptional biomarkers with periodontal clinical indicators

Is dysregulation of the transcriptional biomarkers with optimal classification ability contributing to periodontitis also involved in the progression of periodontitis? We explored this conjecture using the Spearman correlation analysis. Specifically, we calculated the correlation coefficients between the expression of the top three transcripts with the best classification and the measurements or scores of four periodontal clinical indicators (PD, AL, GI, and PI) in 16 periodontitis samples obtained from Jilin University Stomatology Hospital.

### 2.10 Statistical analysis

Before data comparison, normality tests and variance chi-square tests were performed. Student’s t-test was applied for data meeting normal distribution and variance chi-square criteria, Welch’s *t*-test for data satisfying normal distribution but not variance chi-square, and the Mann-Whitney test for data not adhering to normal distribution. In correlation analysis, Pearson correlation analysis was utilized for datasets adhering to the normal distribution, and Spearman correlation analysis was used for those that did not. The above study was completed utilizing SPSS software (version 25.0). In addition, GraphPad Prism (version 9.5) was used for assistance and graphing. Significance levels of *p* < 0.05. Gpower (version 3.1.9.7) calculated the statistical power of each analysis.

## 3 Results

### 3.1 Differentially expressed disulfidptosis- and ferroptosis-related transcripts for periodontitis samples and healthy samples

**FIGURE 1:**
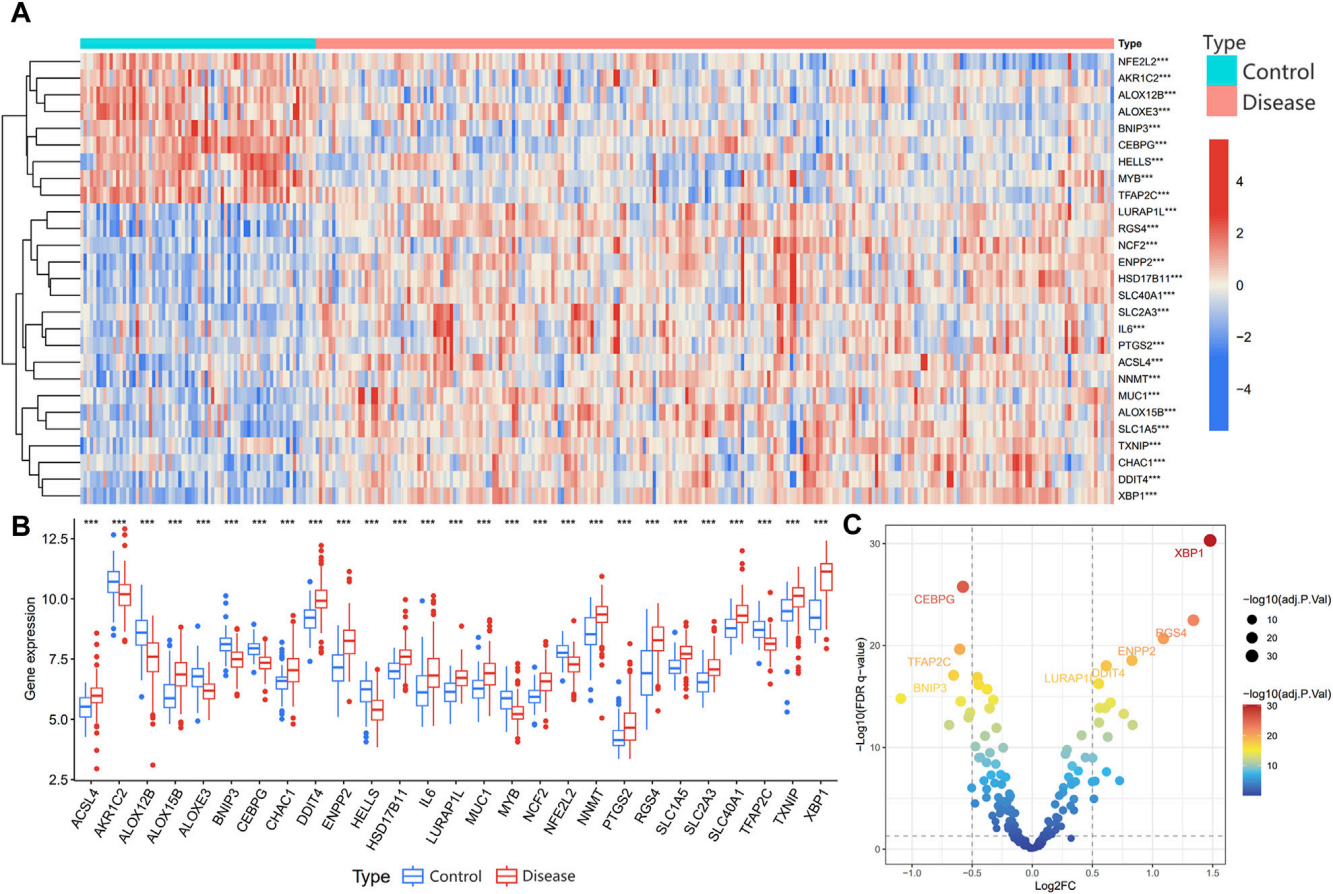
Differentially expressed disulfidptosis- and ferroptosis-related transcripts in periodontitis. **(A)** The heatmap plot showed the expression of 27 transcripts in the periodontitis and control groups. **(B)** The box plot showed the expression of 27 transcripts in the periodontitis and control groups. **(C)** The volcano plot showed that 199 transcripts were differentially expressed between the periodontitis and experimental groups, with 27 transcripts significantly dysregulated. The top ten most significant transcripts were labeled.

23 disulfidptosis related transcripts were sourced from previous literature and the MSigDB website. Ferroptosis related transcripts were sourced from the FerrDb database. After removing duplicate transcripts from three subgroups (drivers, suppressors, and traits), 259 ferroptosis related transcripts were obtained. Detailed information on disulfidptosis related transcripts and ferroptosis related transcripts is provided in Supplementary Material S4. Through Pearson correlation analysis, a total of 199 disulfidptosis- and ferroptosis-related transcripts were identified. Ultimately, 27 differentially expressed disulfidptosis- and ferroptosis-related transcripts were identified: 18 upregulated and nine downregulated. The visualization results of these differentially expressed disulfidptosis- and ferroptosis-related transcripts are displayed in heatmap, box, and volcano plots (Figures 1A–C).

### 3.2 Molecular mechanisms of differentially expressed disulfidptosis- and ferroptosis-related transcripts

**FIGURE 2:**
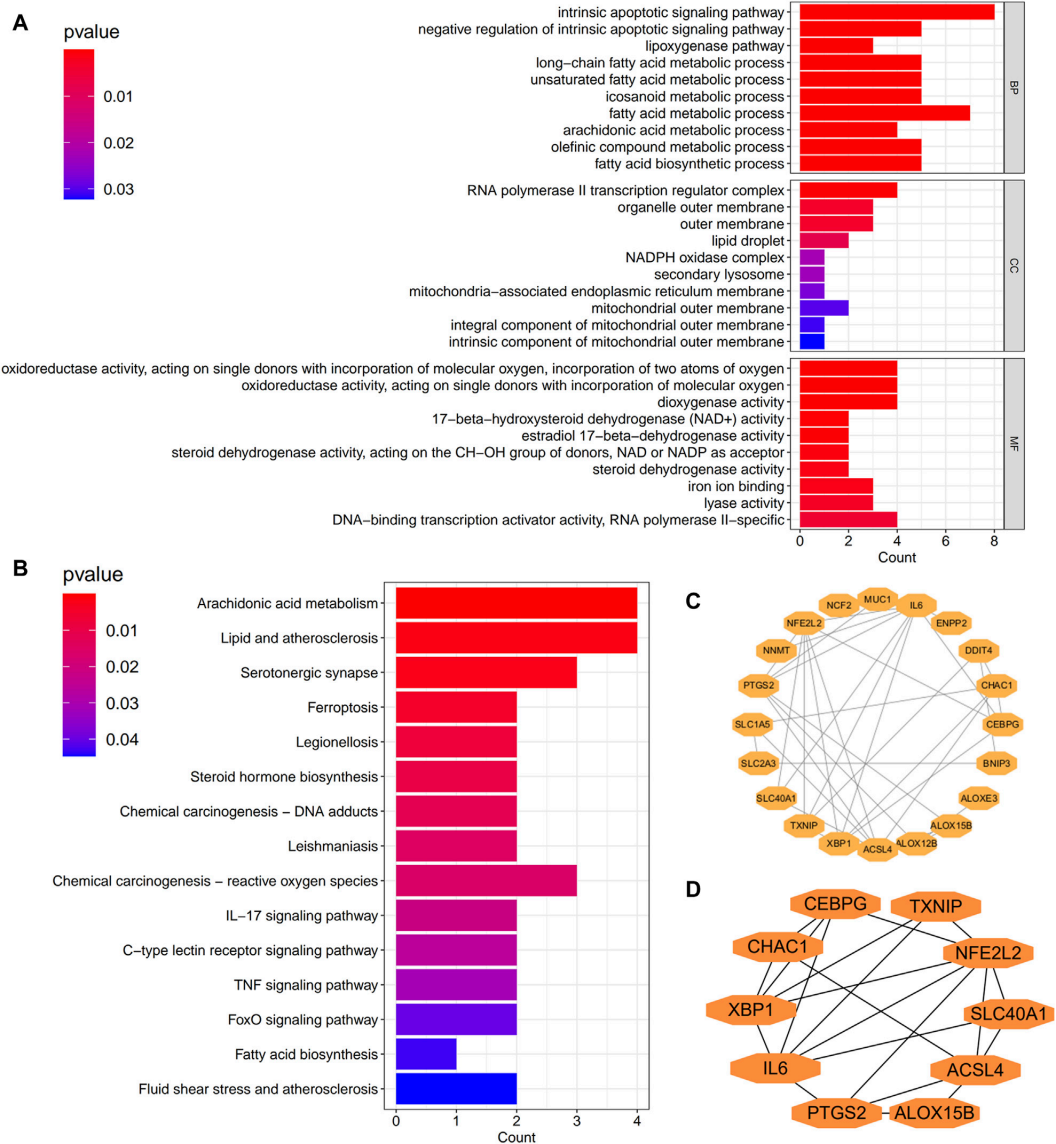
Molecular mechanisms of 27 differentially expressed disulfidptosis- and ferroptosis-related transcripts. **(A)** GO function enrichment analysis. **(B)** KEGG pathway analysis. GO, Gene Ontology; BP, Biological process; CC, Cellular component; MF, Molecular function; KEGG, Kyoto Encyclopedia of genes and Genomes. **(C)** A protein-protein interaction (PPI) network of differentially expressed disulfidptosis- and ferroptosis-related transcripts. **(D)** The top ten protein products of transcripts are at the PPI network’s hub positions.

We explored the molecular mechanisms of differentially expressed disulfidptosis- and ferroptosis-related transcripts. The significant GO-BP (biological process) terms were predominantly related to the intrinsic apoptotic signaling pathway, lipoxygenase pathway, and fatty acid metabolic process. The GO-CC (cellular component) analysis revealed that differentially expressed disulfidptosis- and ferroptosis-related transcripts were primarily enriched in the RNA polymerase II transcription regulator complex, organelle outer membrane, and outer membrane. The GO-MF (molecular function) is associated mainly with oxidoreductase activity, dioxygenase activity, and 17-beta-hydroxysteroid dehydrogenase (NAD+) activity. KEGG analysis indicated that these transcripts were significantly enriched in arachidonic acid metabolism, lipid and atherosclerosis, and serotonergic synapse. The bar plot graphically represented the functional enrichment analysis results, as Figures 2A, B depicts. The differentially expressed disulfidptosis- and ferroptosis-related transcripts related protein-protein interaction network is illustrated in Figure 2C. Upon removal of the isolated nodes, the PPI network comprised 20 nodes and 36 edges. The top ten transcripts at the hub positions are illustrated in Figure 2D.

### 3.3 Machine learning to identify disulfidptosis- and ferroptosis-related transcriptional biomarkers

**FIGURE 3:**
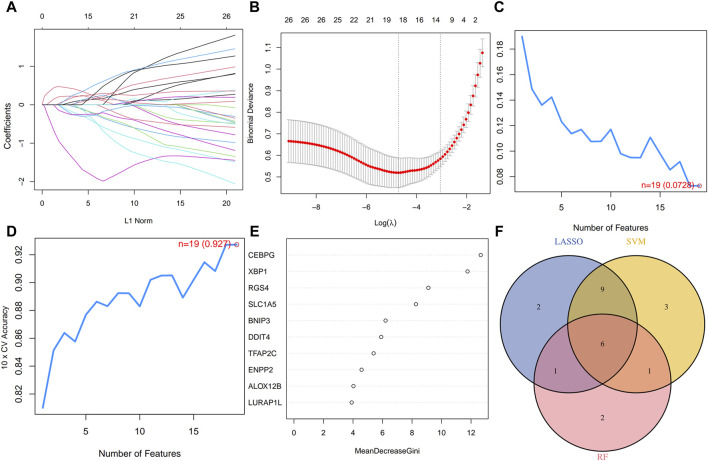
Machine learning to identify disulfidptosis- and ferroptosis-related transcriptional biomarkers. **(A–E)** Construction of disulfidptosis- and ferroptosis-related transcriptional biomarkers using LASSO regression, SVM, and RF algorithm. **(F)** The Venn diagram showed the intersection of results from three machine learning methods. LASSO, least absolute shrinkage, and selection operator; SVM, support vector machine; RF, random forest.

We employed three machine learning methods to further refine the identification of transcriptional biomarkers in periodontitis from differentially expressed disulfidptosis- and ferroptosis-related transcripts: LASSO, SVM, and RF. LASSO regression yielded a total of 18 outputs (Figures 3A, B), SVM identified 19 transcripts (Figures 3C, D), and RF selected 10 transcripts based on the top 10 importance scores (Figure 3E). The results derived from the three machine learning methods were intersected and illustrated in a Venn diagram (Figure 3F), revealing six transcriptional biomarkers critical for differentiating periodontitis tissues from healthy tissues: ALOX12B, BNIP3, CEBPG, LURAP1L, RGS4, and TFAP2C. Among these, LURAP1L and RGS4 demonstrated significant upregulation in expression levels. ALOX12B, BNIP3, CEBPG, and TFAP2C were identified as significantly downregulated transcripts.

### 3.4 Disulfidptosis- and ferroptosis-related transcripts classification model and nomogram model

A logistic regression model was developed to differentiate periodontitis from healthy samples using the six transcriptional biomarkers, calculating a final risk score. The final risk score was computed as follows: (−0.36ALOX12B) + (−1.17BNIP3) + (−3.24CEBPG) + (1.02LURAP1L) + (0.25RGS4) + (−1.61TFAP2C). Based on the expression levels of the six transcriptional biomarkers in each patient, the final risk score for developing the disease can be calculated, providing new perspectives for the early prediction and diagnosis of periodontitis.

**FIGURE 4:**
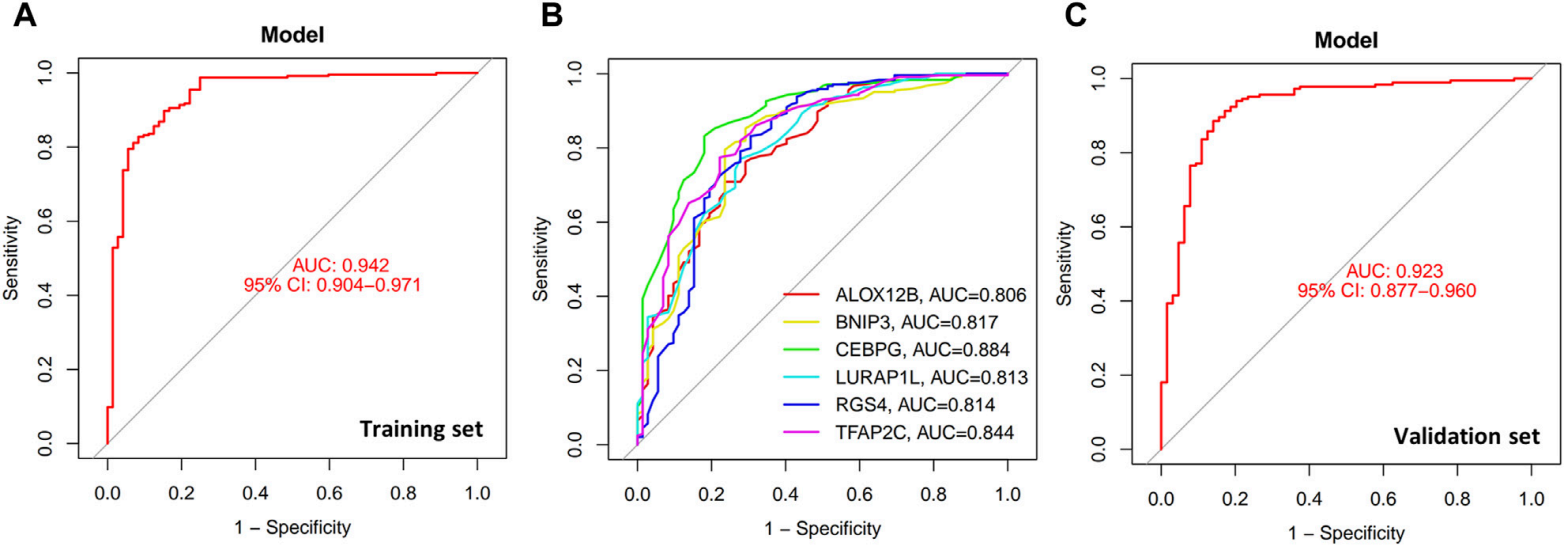
six disulfidptosis- and ferroptosis-related transcriptional biomarkers classification model. **(A)** ROC analysis of classification models for the training set. **(B)** ROC analysis of six transcriptional biomarkers. **(C)** ROC analysis of classification models for the validation set. ROC, receiver operating characteristic.

To further evaluate the accuracy of the model above, we calculated the AUC value of the model as well as the AUC value of each transcriptional biomarker. An AUC value of less than 0.5 represents a poor predictive ability of the model; a value between 0.5 and 0.7 indicates an average predictive ability; a value between 0.7 and 0.9 indicates a good predictive ability; and a value higher than 0.9 indicates that the model has an excellent predictive ability. The AUC value for the training set of the model is 0.942 (95% CI: 0.904-0.971) (Figure 4A), and the AUC value for the validation set is 0.923 (95% CI: 0.877-0.960) (Figure 4C). These results demonstrate that the model has excellent predictive ability. The AUC values of each transcriptional biomarker are shown in Figure 4B. The top three transcripts with the best classification ability were CEBPG (AUC = 0.884), TFAP2C (AUC = 0.844), and BNIP3 (AUC = 0.817). These AUCs were determined using single-transcript models. Therefore, these three could be correlated.

**FIGURE 5:**
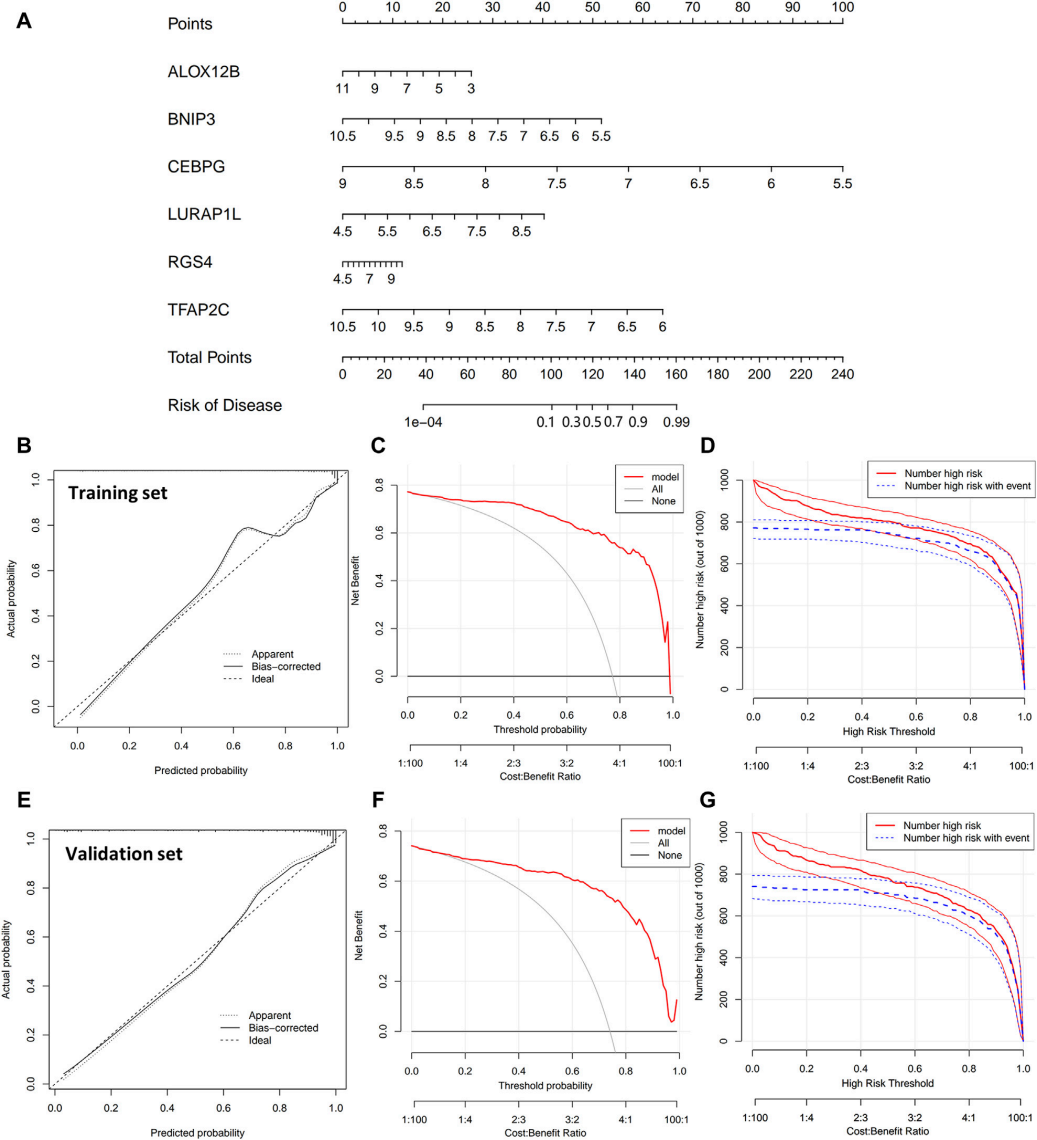
**(A)** The nomogram model based on the six disulfidptosis- and ferroptosis-related transcriptional biomarkers. **(B–D)** The calibration, DCA, and CIC of the nomogram model for the training set. **(E–G)** The calibration curves, DCA, and CIC of the nomogram model are used for the validation set.

A nomogram model (Figure 5A) was also constructed based on the six transcriptional biomarkers, providing an intuitive representation of the impact of each transcriptional biomarker on the disease. In Figure 5A, we can calculate the disease risk in a patient from the expression of six transcripts in that patient. This has profound implications in the early diagnosis of periodontitis. Calibration curves (Figure 5B), DCA (Figure 5C), and CIC (Figure 5D) were evaluated to assess the performance of the nomogram model. Calibration curves are scatter plots about actual and predicted incidence, in which we can see that the predicted values are closer to the exact values. In DCA, the red line represented by the model is on the “All” and “None” lines above. In CIC, the red curve described by the model prediction matches well with the blue curve represented by the actual occurrence. All the above results indicate that the model predictions are good. Additionally, the model was validated using a separate validation set, with the results displayed in Figures 5E–G.

### 3.5 Immune infiltration analysis

**FIGURE 6:**
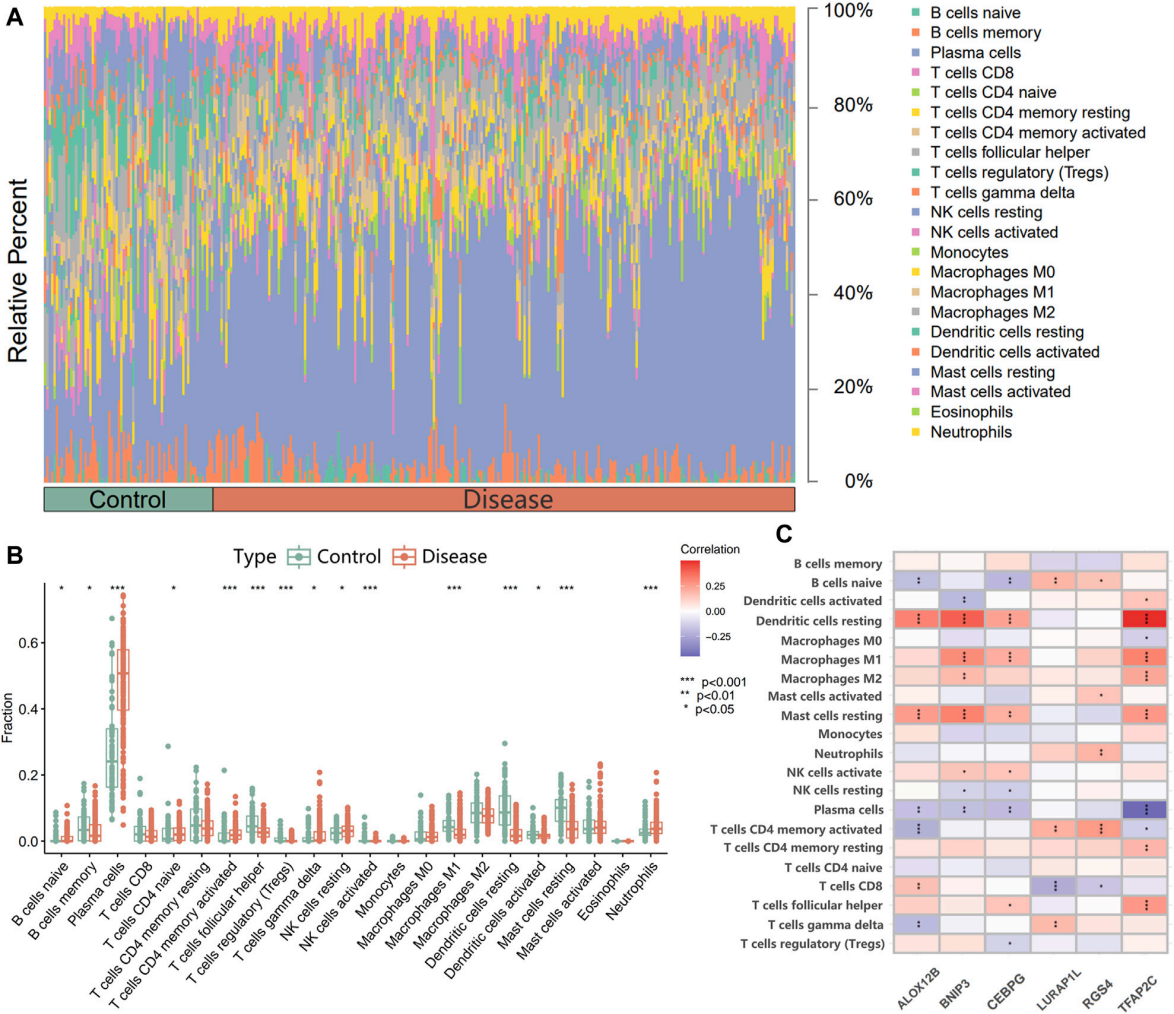
**(A)** Proportion of 22 immune cell infiltrations between periodontitis and control groups. **(B)** Differences in immune cell infiltration between periodontitis and control groups. **(C)** Correlation between six characteristic disulfidptosis- and ferroptosis-related transcriptional biomarkers and infiltrating immune cells.

An analysis of infiltrating immune cells was conducted to investigate the differential characteristics of the immune microenvironment between diseased and healthy groups (Figures 6A, B). In periodontitis samples, compared to healthy ones, the infiltration levels of naive B cells, plasma cells, native CD4^+^ T cells, activated memory CD4^+^ T cells, γδT cells, and neutrophils were significantly elevated. In contrast, those of T follicular helper cells (Tfh) and regulatory T cells (Tregs) were notably reduced. Furthermore, to elucidate the biological relationship between DFR transcripts and the immune microenvironment, a study was conducted on the association between the six transcriptional biomarkers and infiltrating immune cells (Figure 6C). The results showed that the six transcriptional biomarkers were closely associated with multiple immune cells in periodontitis samples, particularly TFAP2C, CEBPG, and BNIP3. Interestingly, these three transcripts were transcriptional biomarkers with the highest classification efficacy in the ROC analysis.

### 3.6 Two disulfidptosis- and ferroptosis-related subclusters for periodontitis

**FIGURE 7:**
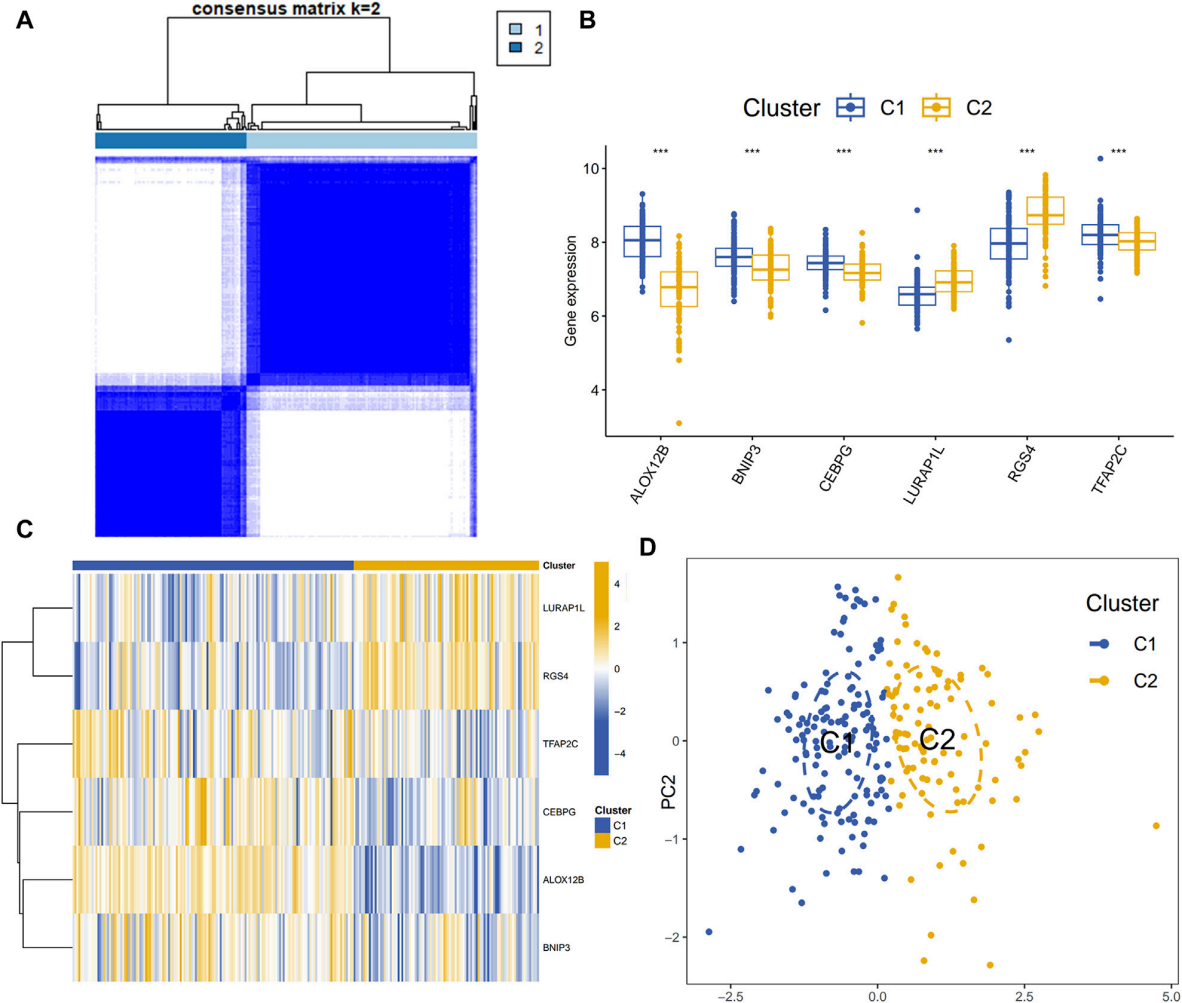
**(A)** Disulfidptosis- and ferroptosis-related subclusters for periodontitis. **(B,C)** Boxplot and heatmap showed differential expression of disulfidptosis- and ferroptosis-related transcripts between subclusters. **(D)** PCA diagram showed the distribution of different subclusters. PCA, principal component analysis.

**FIGURE 8:**
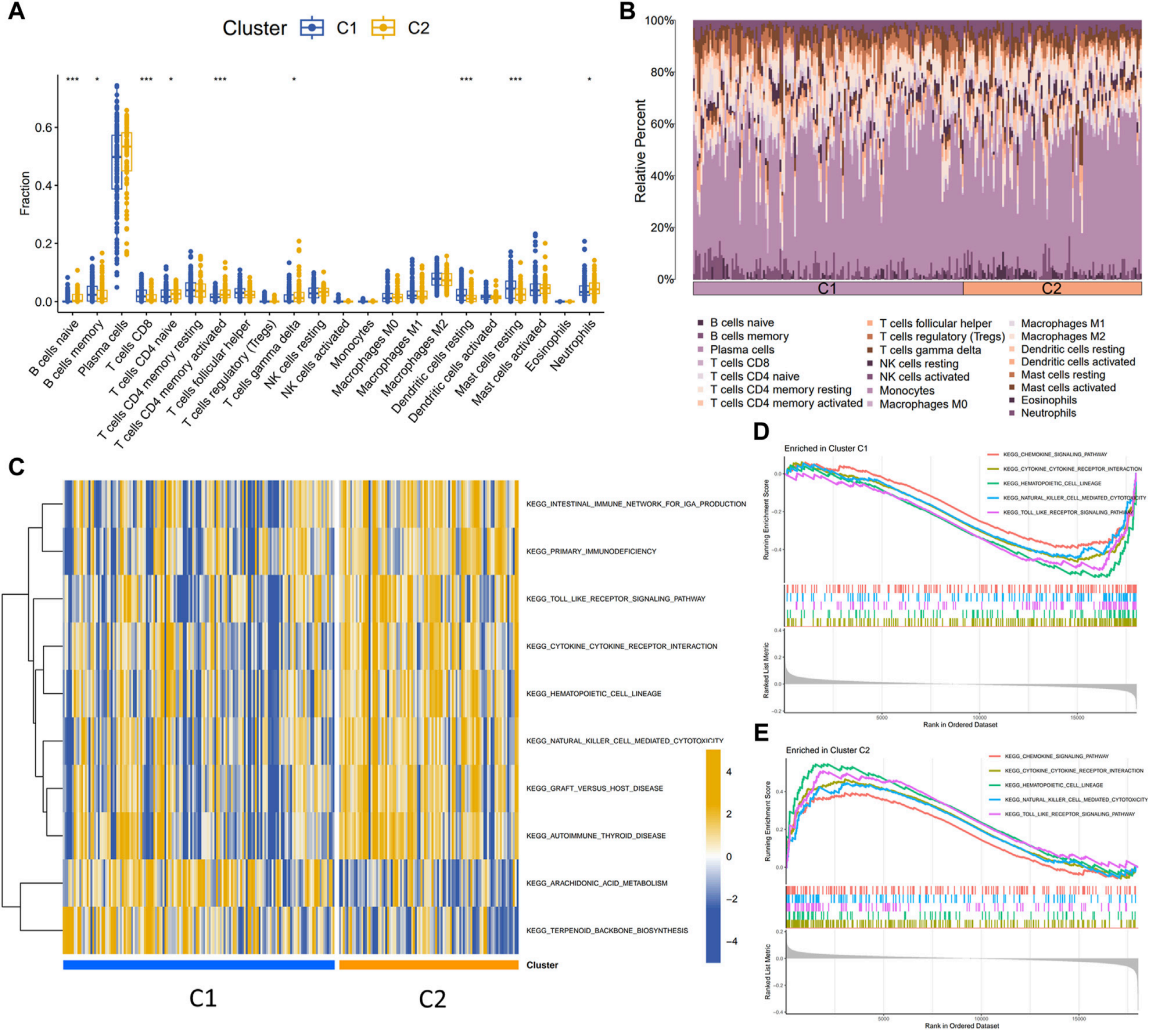
**(A,B)** The variations in immune cell infiltration between the C1 and C2 subclusters. **(C)** GSEA elucidated the functional disparities between disulfidptosis- and ferroptosis-related subclusters. **(D,E)** GSVA elucidated the functional disparities between disulfidptosis- and ferroptosis-related subclusters. GSVA, Gene Set Variation Analysis; GSEA, Gene Set Enrichment Analysis.

An unsupervised consistency clustering analysis of the validation set of periodontitis samples identified two disulfidptosis- and ferroptosis-related subclusters, utilizing the expression profiles of six disulfidptosis- and ferroptosis-related transcriptional biomarkers. Significant variations in the expression of the six transcriptional biomarkers were observed across the subclusters (Figure 7). Figures 8A, B illustrated the variations in immune cell infiltration between the C1 and C2 subclusters. Compared to the C2, the C1 exhibited higher activity of memory B cells, CD8^+^ T cells, resting dendritic cells, and resting mast cells. In contrast, the C2 demonstrated increased activity of naive B cells and activated CD4^+^ memory T cells. GSEA (Figure 8C) yielded similar findings. GSVA (Figures 8D, E) revealed that the chemokine signaling pathway, cytokine-cytokine receptor interaction, hematopoietic cell lineage, natural killer cell mediated cytotoxicity, and Toll-like receptor signaling pathway were active in the C2 isoform and inactive in the C1 isoform.

### 3.7 Clinical experiment validation

**FIGURE 9:**
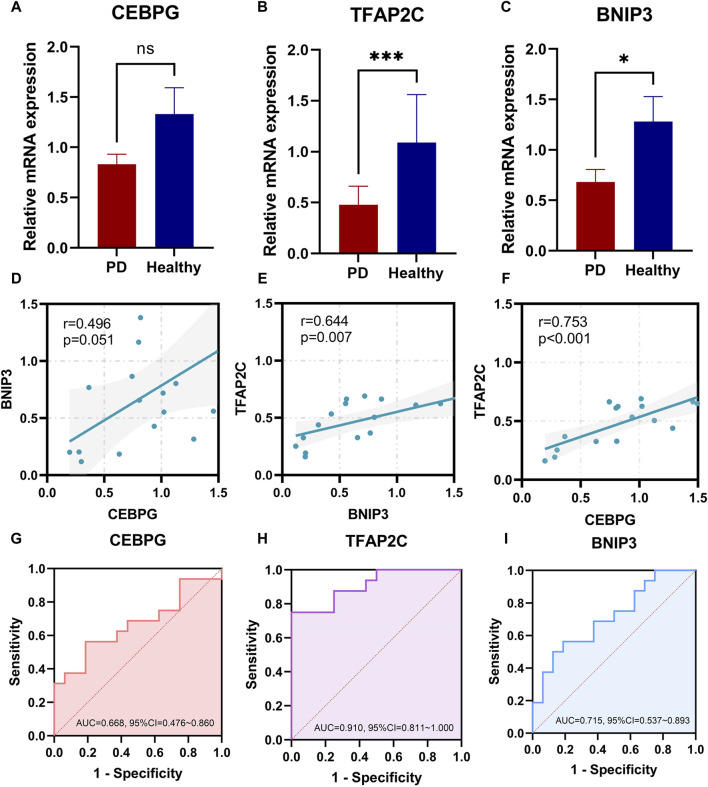
**(A–C)** qRT-PCR validation of the top three transcripts with the best classification (CEBPG, TFAP2C, and BNIP3). (*p < 0.05; **p < 0.01; ***p < 0.001) **(D–F)** The correlation among the top three transcripts with the best classification in 16 periodontitis samples. **(G–I)** ROC curves for 32 study cases of the top three transcripts with the best classification from Jilin University Stomatological Hospital. ROC, receiver operating characteristic.

Validation of the top three transcripts with the best classification ability expression was conducted using quantitative real-time PCR. The results (Figures 9A–C) demonstrated general concordance with the microarray data analysis. The TFAP2C and BNIP3 transcripts exhibited downregulated expression in the periodontitis group and showed significantly lower expression levels than the control subject. However, no significant difference was observed in the expression levels of CEBPG between the periodontitis and healthy groups. Correlation analysis (Figures 9D–F) indicated that the top three transcripts with the best classification ability were positively correlated, suggesting a positive interaction. ROC curve analysis (Figures 9G–I) revealed that CEBPG, TFAP2C, and BNIP3 demonstrated promising efficacy in distinguishing between periodontal inflammatory tissue and healthy tissue, consistent with the findings from the public database analysis.

### 3.8 Correlation analysis between the top three transcripts with the best classification expression and periodontal clinical indicators

The severity of periodontitis was quantified using four periodontal clinical indicators. We calculated Spearman correlation coefficients between the expression levels of the top three transcripts with the best classification and periodontal clinical indicators in 16 patients with periodontitis collected from Jilin University Stomatology Hospital. The analysis results (Table 1) indicated no significant association between the two (*p* > 0.05).

**TABLE 1:** Spearman correlation coefficients between the expression levels of the top three transcripts with the best classification and periodontal clinical indicators.

Periodontal clinical indicators	Spearman correlation coefficients (r)		
	CEBPG	TFAP2C	BNIP3
PD	−0.01	0.10	−0.09
AL	0.07	0.23	0.21
GI	−0.05	0.34	0.21
PI	0.04	0.32	−0.11

## 4 Discussion

Periodontitis, an inflammatory disease, is predominantly caused by plaque microorganisms and results in irreversible damage to periodontal tissues. Multiple regulatory cell deaths, such as cuproptosis, autophagy, pyroptosis, and necroptosis, have been extensively studied in periodontitis (Song et al., 2017b). Yet, the roles of disulfidptosis and ferroptosis in this disease remain to be clarified. Both ferroptosis and disulfidptosis seem to be closely linked to the redox state. Therefore, our study utilized public databases and clinical samples to associate disulfidptosis- and ferroptosis-related transcripts with the pathogenesis of periodontitis through bioinformatics analysis and experimental validation. This approach aimed to identify potential critical transcripts for disulfidptosis- and ferroptosis-related transcripts in periodontal tissues, offering new insights into the pathological role of DFR in periodontitis and its interplay with the immune microenvironment. In this research, we identified 27 disulfidptosis- and ferroptosis-related transcripts with dysregulated expression in periodontitis, six transcripts that are tissue biomarkers for periodontitis, the top three transcripts with the best classification, established correlations between these transcriptional biomarkers and the immune microenvironment, developed a classification model based on the six transcriptional biomarkers, and delineated two disulfidptosis- and ferroptosis-related expression patterns in periodontitis.

Initially, this study identified 27 differentially expressed disulfidptosis- and ferroptosis-related transcripts. GO and KEGG analyses revealed that these transcripts are functionally related to the intrinsic apoptotic signaling pathway, fatty acid metabolic process, and oxidoreductase activity. Enrichment was observed in the NADPH oxidase complex, mitochondria, and other cellular components. NADPH plays an essential part in both disulfidptosis and ferroptosis. The reduced state of NADPH counteracts disulfide stress and prevents cellular disulfidptosis (Zheng et al., 2023). Furthermore, the increased oxidation of NADPH, disruption of cellular redox homeostasis, and altered lipid metabolism constitute important biochemical and metabolic characteristics of ferroptosis (Yang and Stockwell, 2016; Lin et al. 2016). Additionally, morphological characteristics of ferroptosis encompass mitochondrial contraction, enhanced mitochondrial membrane density, and the rupture of cellular and mitochondrial membranes (Xie et al., 2016). This observation aligns with our study’s findings, providing additional evidence that disulfidptosis and ferroptosis could play a role in the development of periodontitis.

Secondly, our study identified six transcripts that are tissue biomarkers for periodontitis (ALOX12B, BNIP3, CEBPG, LURAP1L, RGS4, and TFAP2C) potentially significant for periodontitis using three machine learning methods: LASSO, SVM, and RF. LURAP1L and RGS4 showed significant overexpression, whereas ALOX12B, BNIP3, CEBPG, and TFAP2C were notably underexpressed in periodontitis. CEBPG, also known as C/EBPγ, belongs to the C/EBP family. Since CEBPG is challenging in forming stable homodimers, it usually forms heterodimers with other family members and represses its transcriptional activity. C/EBPγ forms a heterodimer with ATF4 to alleviate various stress responses such as oxidative stress, etc., and acts as a new antioxidant modulator to regulate redox homeostasis (Huggins et al., 2015). TFAP2C is part of the TFAP2 family (transcription factor activating protein 2). The study showed that TFAP2C is a master regulator of periodontitis (Sawle et al., 2016). BNIP3 protein belongs to the Bcl-2 protein superfamily and promotes mitochondrial autophagy (Zhang and Ney, 2009). Mitochondrial autophagy is critical in osteogenesis (Pei et al., 2018). Exposure to inflammatory cytokines inhibits mitochondrial autophagy, suppressing osteogenesis in periodontal ligament stem cells (PDLSCs) (Lin et al., 2023). Specifically, exposure to inflammatory cytokines inhibits mitochondrial autophagy, suppressing osteogenesis in periodontal ligament stem cells (PDLSCs) (Lin et al., 2023). ALOX12B, from the lipoxygenase (LOX) family, catalyzes the formation of hydroperoxides from polyunsaturated fatty acids (Mashima and Okuyama, 2015). ALOX12B is expressed in dermal epithelial cells, and its metabolites are essential for skin barrier protection (Mashima and Okuyama, 2015). RGS4, or Regulator of G-protein Signaling 4, is recognized as a biomarker of ferroptosis (Dixon et al., 2014). Most studies on these transcripts are bioinformatics-based (Xu et al., 2023), and further investigation is needed to elucidate their role in periodontitis. Identifying these periodontitis biomarkers offers new avenues for early monitoring, risk assessment, and disease progression treatment.

Moreover, a classification model was constructed based on the six transcriptional biomarkers, demonstrating higher AUC values than each transcript. This suggests the combined analysis of these six transcriptional biomarkers is more effective in distinguishing periodontitis gingival tissues from healthy ones. Additionally, our study constructed a nomogram model to visualize the contribution of the degree of transcript expression to the disease risk, providing new insights into the prediction of periodontitis risk from a molecular perspective. An external dataset (GSE10334) validated the efficacy of the classification model and nomogram models’ efficacy. We screened the top three transcripts (CEBPG, TFAP2C, BNIP3) with optimal classification ability among these six transcriptional biomarkers and verified their expression, classification efficacy, and correlations through clinical experiments. We collected 32 gingival samples (16 gingival samples from periodontitis patients and 16 gingival samples from healthy patients) at Jilin University Stomatological Hospital. We recorded the information of these subjects, including gender, age, PD, AL GI, and PI. First, qRT-PCR using these gingival tissues helped us to verify the expression levels of the top three transcripts (CEBPG, TFAP2C, BNIP3) with optimal classification ability. The expression levels of BNIP3 and TFAP2C aligned with the analytical outcomes, exhibiting a notably reduced expression compared to the control group. However, the expression of CEBPG showed no significant deviation from the controls. This lack of significant difference may be ascribed to the limited size of the sample group. Second, These AUCs were determined using single-transcript models. Therefore, these three could be correlated. We performed a correlation analysis of these three transcripts using the expression levels of the transcripts obtained by qRT-PCR. The results confirmed our hypothesis that these three could be correlated. Further, the expression of these three transcripts was dysregulated in periodontitis patients compared to gingival tissues from healthy populations. So, is there a correlation between their expression and the severity of periodontitis? Correlating patient clinical information with transcript expression is one of the highlights of this study. Our results showed no significant correlation between these three transcripts’ expression levels and clinical indicators of periodontitis. After excluding the interference of age and gender (Supplementary Material S5), we initially hypothesized that these three transcripts expression levels may be involved only in the onset of periodontitis and may not be significantly involved in the development and progression of periodontitis. However, our speculation still lacks sufficient evidence, and further studies are needed to elucidate this point.

Thirdly, Immune cells, integral to the immune system, are closely linked to the development of periodontitis (Kinane et al., 2007; Kinane and George, 2009). Our study investigated the differences in immune cell infiltration between healthy individuals and periodontitis patients and the correlation between six transcriptional biomarkers and immune cells. Significant alterations in immune cell ratios were observed between healthy individuals and periodontitis patients, with increased levels of naive B cells, plasma cells, native CD4^+^ T cells, activated memory CD4^+^ T cells, γδT cells, and neutrophils in periodontitis tissues. Conversely, the infiltration levels of Tfh and Tregs were significantly reduced. This is consistent with existing studies (Li et al., 2020). Furthermore, Spearman correlation analysis revealed correlations between six transcriptional biomarkers and immune cells. Among the six transcriptional biomarkers, the top three transcripts with the best classification (BNIP3, CEBPG, and TFAP2C) exhibited a closer association with immune cells. Specifically, dendritic cells, M1 macrophages, and resting mast cells showed positive correlations with these transcripts, while plasma cells demonstrated negative correlations. This suggests that the top three transcripts with the best classification possess strong classification efficacy and may have an essential role in influencing the pathogenesis of periodontitis through immune functions. In the array of cells infiltrated by periodontitis, B cells, and plasma cells predominantly occupy the inflammatory tissue (Zouali, 2017). B cells have a dual role in PD, promoting bacterial clearance and the destructive effects of inflammation, bone resorption, and matrix lysis (Berglundh, Donati, and Zitzmann, 2007). Plasma cells, which develop from B cells, are characterized by their high number and density, marking a critical distinction between advanced periodontitis and long-term gingivitis without attachment or bone loss (Thorbert-Mros et al., 2015). Naive CD4^+^ T cells become activated and differentiate into various subtypes upon interaction with antigenic complexes (Luckheeram et al., 2012). Among the subtypes, CD4^+^ T cells are the primary source of IL-17, a cytokine closely associated with periapical bone loss (Luckheeram et al., 2012). An imbalance between Th17 cells and Tregs plays a critical role in periodontitis, with Th17 cells related to inflammation and destruction of periodontal tissues (Monasterio et al., 2018), and Tregs implicated in reducing bone resorption in periodontitis (Cafferata et al., 2021). γδT cells are more prevalent in inflamed gingiva than in healthy individuals, consistent with our study (Lundqvist et al., 1994; Kawahara et al., 1995). Previous evidence suggests a potential role for γδT cells in periodontitis. Neutrophils have increasingly been recognized for their irreplaceable role in periodontitis (Herrero-Cervera et al., 2022). Besides releasing inflammatory mediators with both pro-inflammatory and anti-inflammatory effects, neutrophils also release toxic substances like ROS and collagenase, which contribute to connective tissue damage and the initiation of bone resorption (Lee et al., 1995; Chapple and Matthews, 2007). The correlation of disulfidptosis- and ferroptosis-related transcriptional biomarkers, especially the top three transcripts with the best classification, with various immune cells, disulfidptosis and ferroptosis were associated with immune responses. Still, the exact mechanism needs to be further investigated.

Fourthly, using unsupervised consistent clustering analysis, two periodontitis subclusters (C1 and C2) were identified based on differing expression patterns of the six characterized DFRs. In the C1 subcluster, ALOX12B, BNIP3, CEBPG, and TFAP2C showed higher expression, while LURAP1L and RGS4 were more expressed in the C2 subcluster. Additionally, these subclusters exhibited distinct immune infiltration profiles and functional pathways. Immune cells that were more infiltrated in periodontitis were more infiltrated in the C2 than in the C1. In addition, the results of GSEA and GSVA showed that C2 was more enriched in immune-related signaling pathways. Cytokines, critical peptide mediators for cell signaling and communication, perform various functions, including modulating immune and inflammatory responses (Ramadan et al., 2020). Chemokines coordinate leukocyte recruitment and activation, thus attracting macrophages, neutrophils, and lymphocytes to inflammation sites (Ramadan et al., 2020). The inflammatory process involves a complex network of cytokines and chemokines, crucial in mediating inflammation in periodontal tissue (Ramadan et al., 2020). Hematopoietic cell lineage encompasses various blood cells differentiating from hematopoietic stem cells, serving as the foundational source of immune cells. Toll-like receptors (TLRs) recognize pathogens and play a pivotal part in activating the host’s innate immune response and adaptive immunity against periodontal disease bacteria (Song et al., 2017a). In summary, the C2 subcluster demonstrates greater immune relevance than the C1 subcluster. This clustering approach, widely utilized in oncology research, has significantly contributed to the clinical management of tumors and clinical decision-making. Identifying various disulfidptosis- and ferroptosis-related subtypes enhances understanding of the role of disulfidptosis and ferroptosis in periodontitis and offers new insights into prognostic assessment and personalized treatment. Owing to limited clinical data in the public database, further investigation of this molecular-level typing about clinical symptoms and prognosis of periodontitis patients is necessary, potentially paving the way for its future application in clinical decision-making and treatment.

This study represents the first instance of linking disulfidptosis to ferroptosis and exploring their pathophysiological roles in periodontitis and their interactions with the immune microenvironment. This work has led to several novel discoveries that may inform future research on the relationship between cell death mechanisms and periodontitis. However, the current study does have some limitations. Although the expression of disulfidptosis- and ferroptosis-related transcripts with the best classification efficiency was validated in a clinical trial, further validation with a larger sample size is needed. Additionally, the absence of comprehensive clinical data, including symptoms and prognostic information in public databases, limited our ability to probe further the association between disulfidptosis- and ferroptosis-related transcripts -based periodontitis subtypes and clinical features. It is essential to elucidate the precise molecular pathways involved in disulfidptosis and ferroptosis within the context of periodontitis in the future to advance understanding in this field.

## 5 Conclusion

Our work integrates disulfidptosis and ferroptosis for the first time, utilizing bioinformatics approaches to investigate their role in periodontitis. We constructed a classification model incorporating six disulfidptosis- and ferroptosis-related transcriptional biomarkers, and consistency clustering analysis yielded two disulfidptosis- and ferroptosis-related subtypes enhances understanding of the role of disulfidptosis and ferroptosis in periodontitis and offers new insights into prognostic assessment and personalized treatment modification patterns. Our study demonstrated that six transcripts that are tissue biomarkers for periodontitis, particularly the top three transcripts with the best classification, exhibit correlations with multiple infiltrating immune cells. This information indicates that disulfidptosis and ferroptosis could affect periodontitis by eliciting immune responses. Additionally, the top three transcripts with the best classification we discovered may be involved only in the onset of periodontitis and may not be significantly involved in the development and progression of periodontitis. This will offer new insights into the mechanisms of disulfidptosis and ferroptosis in both the onset and development of periodontitis.

## Data Availability

Publicly available datasets were analyzed in this study. This data can be found here: GEO database: GSE16134 GSE10334 GSE23586.
