# Supplementary material for: Identification of disulfidptosis- and ferroptosis-related transcripts in periodontitis by bioinformatics analysis and experimental validation
Source: Front Genet. 2024 Jul 9;15:1402663. doi: 10.3389/fgene.2024.1402663 (PMC11263038; doi:10.3389/fgene.2024.1402663)
Supplement: Supplementary file 1 [file DataSheet1.docx]

Supplementary S1

**Inclusion and exclusion criteria:**

**Inclusion criteria:**

**(1) Periodontal health group:**

① Age between 20~60 years old;

② Maximum PD of the whole mouth ≤3 mm and no CAL;

③ BOP (-).

**(2) Periodontitis group:**

① Age between 20~60 years old;

② PD ≥ 4 mm and AL ≥ 4 mm more than 30% of the site.

③ BOP (+).

**Exclusion criteria:**

①History of smoking.

② having major systemic diseases, such as cancer, cardiovascular disease, etc.

③ Being in pregnancy or lactation.

④ Abnormal mental status or poor compliance.

⑤ Have a history of periodontal-related treatment within the last six months.

⑥ Those who have explained antihistamine or anti-inflammatory drug treatment within the last six months.

Supplementary S2

1, PD: PD stands for the distance from the base of the gingival sulcus to the gingival margin, which is measured using a periodontal probe at a total of 6 loci in the proximal-middle, intermediate, and distal sides of the labial/buccal and lingual/palatal sides of each tooth, and the average value is taken. Unit: mm

2, AL: AL represents the PD value minus the distance from the bottom of the periodontal pocket to the cemento-enamel junction. Measurements were taken using a periodontal probe at a total of 6 sites in the labial/buccal and lingual/palatal sides of each tooth in the proximal-medial, intermediate and distal regions, and the average value was taken. Unit: mm

3, PI: 0 points represents that no plaque was detected; 1 represents that a small amount of plaque exists in the gingival margin area, which is not visible to the naked eye but can be scraped out by the probe; 2 represents that the adjacent surfaces of the teeth at the gingival margins have a moderate amount of plaque; and 3 represents that there is a large amount of plaque at the gingival margins, in the gingival sulcus, or on the adjacent surfaces of the teeth.

4, GI: 0 points represents healthy gums; 1 points represents mild inflammation of the gingiva, mild swelling and color change, BOP (-); 2 points represents moderate inflammation of the gingiva, gingival swelling and shiny, reddish color, BOP (+); 3 points represents severe inflammation of the gingiva, gingiva is obviously swollen or ulcerated, there is spontaneous bleeding.

Supplementary S3

|  | Forward primer | Reverse primer |
| --- | --- | --- |
| GAPDH | CACCCACTCCTCCACCTTTGAC | GTCCACCACCCTGTTGCTGTAG |
| CEBPG | ACTCCAGGGGTGAACGGAAT | CATGGGCGAACTCTTTTTGCT |
| BNIP3 | TGAGTCTGGACGGAGTAGCTC | CCCTGTTGGTATCTTGTGGTGT |
| TFAP2C | TCAGTCCCTGGAAGATTGTCG | CCAGTAACGAGGCATTTAAGCA |

**Gene primer sequences**

GAPDH was used as an internal control gene.

Supplementary S4

**DRGs:**

| SLC7A11 | GYS1 | NDUFS1 | NDUFA11 | NUBPL | NCKAP1 | LRPPRC |
| --- | --- | --- | --- | --- | --- | --- |
| RPN1 | ACTN4 | ACTB | CD2AP | CAPZB | DSTN | FLNA |
| INF2 | IQGAP1 | MYH10 | MYL6 | MYH9 | PDLIM1 | TLN1 |
| SLC3A2 | FLNB |  |  |  |  |  |

**FRGs:**

| PTGS2 | DUSP1 | NOS2 | NCF2 | MT3 | UBC | ALB |
| --- | --- | --- | --- | --- | --- | --- |
| SRXN1 | GPX2 | BNIP3 | OXSR1 | SELENOS | ANGPTL7 | CHAC1 |
| DDIT4 | LOC284561 | ASNS | TSC22D3 | DDIT3 | JDP2 | SESN2 |
| PCK2 | TXNIP | VLDLR | GPT2 | PSAT1 | LURAP1L | SLC7A5 |
| XBP1 | ATF3 | SLC3A2 | CBS | ATF4 | ZNF419 | KLHL24 |
| ZFP69B | ATP6V1G2 | VEGFA | GDF15 | TUBE1 | ARRDC3 | CEBPG |
| RGS4 | BLOC1S5-TXNDC5 | LOC390705 | EIF2S1 | KIM-1 | IL6 | CXCL2 |
| HSD17B11 | AGPAT3 | SETD1B | HMOX1 | TF | FTL | RPL8 |
| TFRC | MAFG | IL33 | FTH1 | SLC40A1 | GPX4 | HAMP |
| NFE2L2 | STEAP3 | DRD5 | DRD4 | MAP3K5 | MAPK14 | SLC2A1 |
| SLC2A6 | SLC2A8 | SLC2A12 | GLUT13 | SLC2A14 | EIF2AK4 | ALOX5 |
| ALOX15 | ACSF2 | IREB2 | HMGB1 | ELAVL1 | TFAP2C | SP1 |
| NNMT | PLIN4 | HIC1 | STMN1 | RRM2 | CAPG | HNF4A |
| YWHAE | GABPB1 | AURKA | MIR4715 | RIPK1 | PRDX1 | MIR30B |
| AKR1C2 | AKR1C3 | RB1 | HSF1 | GCLC | SQSTM1 | NQO1 |
| MT1G | CISD1 | FANCD2 | FTMT | HSPA5 | TP53 | HELLS |
| FADS2 | SRC | STAT3 | PML | MTOR | NFS1 | TP63 |
| MIR137 | ENPP2 | VDAC2 | FH | CISD2 | MIR9-1 | MIR9-2 |
| ISCU | ACSL3 | OTUB1 | CD44 | LINC00336 | BRD4 | PRDX6 |
| NF2 | ARNTL | HIF1A | JUN | CA9 | TMBIM4 | PLIN2 |
| Fer1HCH | AIFM2 | LAMP2 | ZFP36 | PROM2 | CHMP5 | CHMP6 |
| GCH1 | CS | EMC2 | NOX1 | CYBB | NOX3 | NOX4 |
| DUOX1 | DUOX2 | G6PD | PGD | PIK3CA | FLT3 | SCP2 |
| LPCAT3 | NRAS | KRAS | HRAS | TFR2 | SLC38A1 | SLC1A5 |
| GOT1 | CARS1 | KEAP1 | ATG5 | ATG7 | NCOA4 | ALOX12B |
| ALOXE3 | PHKG2 | ACO1 | G6PDX | ULK1 | ATG3 | ATG4D |
| MAP1LC3A | GABARAPL2 | GABARAPL1 | ATG16L1 | WIPI1 | WIPI2 | SNX4 |
| ULK2 | SAT1 | EGFR | MAPK3 | MAPK1 | BID | ZEB1 |
| CDKN2A | PEBP1 | SOCS1 | CDO1 | MYB | MAPK8 | MAPK9 |
| PRKAA2 | PRKAA1 | BAP1 | ABCC1 | MIR6852 | ACVR1B | TGFBR1 |
| HILPDA | IFNG | ANO6 | LPIN1 | TNFAIP3 | TLR4 | ATM |
| EGLN2 | MIOX | TAZ | MTDH | IDH1 | SIRT1 | FBXW7 |
| DNAJB6 | BACH1 | LONP1 | LINC00472 | EPAS1 | YY1AP1 | PANX1 |
| TXNRD1 | SLC7A11 | SLC1A4 | HERPUD1 | TRIB3 | SNORA16A | RELA |
| ATP5MC3 | HSPB1 | SLC2A3 | ALOX12 | HBA1 | NGB | AKR1C1 |
| MUC1 | SCD | CDKN1A | MIR9-3 | MIR17 | MIR212 | CAV1 |
| NOX5 | ACSL4 | GLS2 | ALOX15B | BECN1 | ATG13 | DPP4 |

Supplementary S5

|  | PD | Health | P |
| --- | --- | --- | --- |
| Age | 46.56±14.04 | 38.94±6.981 | >0.05 |
| Gender | Female: 5 | Female: 8 |  |
|  | Male: 9 | Male: 7 |  |
| PD | 5.25±1.39 | 1.75±0.68 | <0.05 |
| AL | 3.06±1.73 | 0.00±0.00 | <0.05 |
| GI | 2.25±0.58 | 0.19±0.40 | <0.05 |
| PI | 2.13±0.72 | 0.69±0.60 | <0.05 |
